# Dr. Henderson's Observations on the Case of Ann Moore

**Published:** 1813-02

**Authors:** A. Henderson

**Affiliations:** Golden-square


*To the Editors of the Medical and Physical Journal.*

GENTLEMEN,

SOME account of Ann Moore, known by the appellation of the *Fasting-Woman* of Tutbury, having already appeared in your Journal, accompanied by certain speculations on her case, I am induced to transmit to you the particulars of a visit, which I had the curiosity to pay her, with my friend Mr. Lawrence, and another gentleman, when passing through Staffordshire, last summer; and to add a few remarks, with a view to exhibit her true character, and to undeceive the public in regard to her.

Previously to our visit, we had endeavored to collect the opinions of the neighbourhood, concerning this case of alleged extraordinary abstinence. Of the medical gentlemen to whom we addressed ourselves, the majority seemed sceptical on the subject; though it did not appear that any very decisive means had been used by them to prove the fact of imposture: but, among the common people, there was the most implicit belief in the truth of the story; and, whenever we ventured to express any doubts, we were invariably referred to the watching, to which Ann Moore had been subjected, as a full and satisfactory refutation of our incredulity.

On our arrival at Tutbury, we lost no time in proceeding to the dwelling of Ann Moore, whom we found sitting up in a bed so constructed as scarcely to admit of her using the recumbent posture. She did not seem in the least discomposed by our abrupt entrance; though, on reaching the house, some bustle was heard in the upper story, as if preparations had been making for our reception. From the appearance of her countenance, which was natural, and even healthy, and from that of her upper limbs, abdomen, and back, which we examined very carefully, she might be called rather thin; but many persons of her age, in perfect health, are much thinner. The abdomen was *not* contracted, nor did it present any peculiar appearance; nor was the pulsation of the aorta more distinctly perceptible than it is in the generality of persons. The lower extremities, however, seemed, to a certain extent, wasted and paralytic; the pulse was 94, firm and regular; both the hands and feet were moist; her mouth, as far as we were permitted to examine it, shewed no deficiency of saliva; and, on holding a mirror before her face, it was immediately covered with copious moisture. She spoke to us in a distinct and tolerably strong voice, and moved her arms and fingers with considerable force. There was an offensive urinous smell about the bed.

In answer to the questions we put to her, she told us that, on the 31st of October, she would be just 51 years old; that she had tasted no solid food for upwards of five years, and no drink for nearly four years, and had no desire for either; that she never even wetted her lips, except when she washed her face, which happened about once a-week; that she had voided no urine since the week before Easter three years, and no fœces since that day (August 3) five years; that she had not slept or lain down in bed for more than three years; that she sometimes dozed, with her head reclining on the pillow, but never so as to forget herself; that she had frequently blisters applied to the back of her neck, on account of a giddiness in her head, and that they rose and discharged plentifully; but that, in general, she did not experience much uneasiness, nor feel pain, except on pressure of the left hypochondrium; that when she took snuff, which she did habitually, it produced a flow of mucus from the nostrils; that her hands were generally moist; and that she perspired freely over the whole surface of the body when she had fits. The nature of these fits she did not explain. Her mouth, according to her own declaration, she was unable to open, because it occasioned severe pain behind the jaws; but the lower jaw acted freely enough within the sphere in which she chose to move it in our presence, to shew that there was nothing defective in the articulations; the masseter and temporal muscles were soft, and could not, therefore, resist its descent: besides, it was evident, when she spoke, that she could separate her teeth to some extent, and that without giving any indications of uneasiness. Of all the fingers of the left hand, except the index, she said that she had lost the use; the middle finger, indeed, she admitted, could be moved by external force, though not by volition. But while Mr. Lawrence was examining the spot, where she complained of pain on opening her mouth, she was observed to use the finger in question without any difficulty. On attempting to raise the two remaining fingers, which were bent, she made some resistance, and complained of my hurting her. The left hand, she affirmed, was hotter than the other.

Among the circumstances that tend to invalidate many of the above assertions, and, in particular, the statement respecting her prolonged abstinence, the following may be mentioned:

1. *The natural and healthy appearance of her face.*

2. *The strength of her pulse, muscles, and voice.*

3. *The moisture of her mouth, nostrils, eyes, and whole surface of the skin.*—If the functions of the stomach were entirely entirely suspended, or even materially deranged, it is not likely that the saliva would continue to be regularly supplied, since the flow of it depends so much on the healthy state of that organ; and, without taking into account the occasional discharges from blisters and other causes, it is obvious, that the exhalation from the lungs and surface of the body, which we ascertained beyond the possibility of doubt, must occasion a correspondent drain from the internal parts. But these excretions have been found to amount, in a healthy person, to more than 65 ounces in the day. If we allow them to be reduced, by disease, to one half that quantity, it is evident that they would still be sufficient to consume the whole substance of Ann Moore in a very few weeks.

4. *The entireness of her intellectual faculties.*—Long-continued fasting has always been reckoned among the predisposing causes of insanity. In a case recorded by Tulpius, delirium supervened on the twelfth day. The same occurrence took place in that related by Dr. Currie, though a considerable portion of nourishment had been conveyed into the system by means of injections. And, in the "Remarkable Case" described by Dr. Willan, in the second volume of the Medical Communications, much confusion of mind was observable in the latter periods of the disorder.

5. *Her "notorious immoral character," in the former part of her life, and her own confession, that she once,* THROUGH IMPOSITION, *passed for a religious person, merely for the sake of worldly gain."* *—These are strong objections to the reception of her testimony, whatever degree of "calmness and serenity" may mark "her present state of mind;" however "clear and unimpeachable her doctrinal knowledge" may now prove, and however "pleasing it must be to every lover of religion to converse with her." If she once played the hypocrite "for the sake of worldly interest," the presumption is, that she would not scruple to act that or any other part again, from the same motive.

6. *The obvious interest which she and her attendants have in supporting the deception.*—From the published accounts of her case it appears, that, before she began to attract the attention of the public, she had been "laboring under the greatest distresses," and "had not even sufficient clothes to cover her bed:"† but, when we saw her, she seemed to be in very comfortable circumstances; and we were informed by a gentleman of the place, that she has

---

* Account of the Extraordinary Abstinence of Ann Moore. By J— L—. 8vo. Uttoxeter, 1809, page 8.

† Account, &c. p. 16.

 turned turned the exhibition of her person to such account, as to be able, in the course of the present year, to place the sum of 400*l*. in the stocks.

7. *The imperfect manner in which she has been watched.*— As much stress has been laid on the proof afforded by the watching of her person during sixteen days, it may be worth while to inquire in what manner it was performed. The author of the "Account" tells us, that, Ann Moore "having consented to be removed, Mr. Taylor (a surgeon of the neighbourhood) went round the town to procure a number of the most respectable inhabitants for the watch; and he made it his first principle, to expunge those who, in his *opinion*, were in the least degree liable to be imposed on, or of a disposition that might be suspected would connive at imposture. He admitted no persons but such as most vehemently objected to the verity of the fact." "Mr. H. Jackson," we are further assured, "having a *thorough knowledge* of the inhabitants, took upon himself the trouble of setting the watch; and he, being of the most invincible incredulity, was well qualified for the purpose. When it was known that Nanny had been under watch for forty hours, and was challenging the investigation, *great numbers of people*, merely from curiosity, *came to offer their service*, so that there was not the least difficulty in procuring a sufficient number for the purpose. The principal care that remained to Mr. Jackson, was the matching of people of different qualifications together, in such a manner as to afford a greater security, and that the watch should be constantly and faithfully kept. In order to which, such as man and wife, brother and sister, &c. were never suffered to attend at the same time, nor any persons that were likely to be influenced in her favour. The watch was generally changed every four hours, and, for further satisfaction, placards were stuck up in different parts of the town, announcing, 'This is to maintain, that Ann Moore has taken no nourishment since Tuesday afternoon, at three o'clock, and is truly and constantly watched. All persons are hereby challenged to disprove the fact, and may watch for themselves, during the further period of time that shall, by medical consultation, be determined to establish the same.*' " The truth is, that almost every one, who "came to offer" his service, was permitted to undertake the task; and, during the sixteen days that the watch continued, not

* Loc. cit. p. 21, 22.

fewer, fewer, as we were credibly informed, than from 80 to 90 different persons officiated. Among this number, is it not highly probable, that there may have been some of Ann Moore's private friends, who supplied her with food, and connived at her eating and drinking? We are not told what were the respective characters of the individuals employed; whether they were persons of known probity and veracity; no security is given for their vigilance; no information is afforded as to the mode in which they were superintended; but we are called upon to place unlimited confidence in Mr. Taylor's "opinion" of their liability to be imposed upon, in Mr. Jackson's "knowledge" of their characters, and his skill in "matching them together;" in other words, we are called upon to believe an improbable fact upon the most suspicious of all testimony, upon the *opinion* which A has of the dispositions of B, C, D, E, &c. and of the *knowledge* and *care* which X has displayed in *matching* B with D, C with E, and so on. Can any thing be more unsatisfactory? But, should we grant, for a moment, that the proof was as perfect as it was possible for it to be, and that this trial of Ann Moore was conducted with the utmost strictness and regularity,—what would the inference amount to? Why, that she had fasted sixteen days and nights, a period of time during which it is certainly not impossible that she may have endured the privation; not that she has lived five whole years and odd months without any nutriment whatever.

That the human body can be brought to subsist, for a considerable time, on very small quantities of food, has been long known; and, if we consult the records of medicine, we shall find, that there are not wanting well-authenticated instances of even more protracted abstinence than was exhibited on the occasion in question. Thus Doebel gives the history of a hypochondriac, who fasted during a period of 40 days, but died soon after his return to food. Morgagni, on the authority of Fantonus, mentions the instance of a woman, who obstinately refused to take any sustenance, except twice, during 50 days, at the end of which period she died. In the case which is detailed by Dr. Eccles, in the fifth volume of the Edinburgh Medical Essays, the patient continued first 34 days, and afterwards 54 days, without either eating or drinking. It is true, that, during part of this time, nourishing injections were used; but, for the last 20 days of the second fast, even that mode of supply was cut off, and the abstinence was complete in every sense of the word. In Dr. Willan's case, the patient persevered in the disuse of solid food till the 61st day, taking only small quantities of water slightly flavoured with juice of oranges, to moisten his mouth. These two last cases also terminated fatally. The madman, mentioned by Pouteau,* lived 47 days, without taking any thing but a pint and a half of water in the day, and stood constantly in the same position during 38 days of that time: the return to food was followed by a temporary cure of his insanity.

That Anne Moore did not altogether refrain from drinking during the watch of 16 days, is admitted even by Mr. Taylor. "In the course of the first three days of the investigation," says that gentleman, "she swallowed, in the whole, about ℥iss of water; but happening to step into the room while she was swallowing it, the extreme misery of deglutition, and the violent rising of wind resisting its passage to a degree that almost seemed to threaten suffocation, induced me to dissuade her from taking any more, while the experiment that was to vindicate her veracity continued."† The only fact, therefore, that can be learned from this imperfect trial is, that Ann Moore was not seen taking any *solid* food during the space of 16 days and nights. Before that time, it is acknowledged, that "she had *abundant* opportunities" of eating; and since the watching, which has so unaccountably allayed all suspicions with regard to her proceedings, she can be at no loss to procure sufficient aliment, from the quantity that is introduced into the house for her daughter, and the other female who lives with her.

8. *Her dread of a repetition of the watching.*—On Mr. Thompson's proposing to her a second watching, she said, "that she had been upon her *trial* once, which she would not then have submitted to, but to oblige the *minister*, and for nobody in the world would she undergo a repetition of it. Her attendant," Mr. T. adds, "who is as well-educated a hypocrite as her mistress, was pleased to style it, '*a trial for her life.*‡' "

9. *Her dread of all experiments whatever.*—Thus, on one occasion, she refused to allow Dr. D. to hold a mirror before her face, in order to examine her respiration; exclaiming, "No more experiments for me! I have suffered enough already from experiments." At another time, she contrived to break a thermometer, which a gentlemen had put into her hand, in order to ascertain the heat of her body. It is probably from a similar motive, that she now keeps her mouth shut; apprehending, either that her visitors might

* Memoires de l'Academie des Sciences, 1769.

† Med and Phys. Jour. Vol. XX.

‡ Med. and Phys. Jour. Vol. XXIV. p. 312.

put put her powers of deglutition to the test, or that the mere inspection of her tongue might prove the recent introduction of food.

10. *Her concealment of the evacuation of urine.*—At the time of the watching, it is admitted that she passed urine to the amount of a pint in every two days: she has since found it convenient gradually to diminish the quantity, till at last she voids none at all. But there were several pretty strong presumptive proofs of the falsity of this assertion. One of us, in approaching her bed, happened to overturn an utensil which was placed under it, obviously for her use, and which was partly filled with urine. There was also, as I have before remarked, an offensive urinous smell about the bed, to lessen the perceptibility of which is probably the reason of her insisting upon the window being kept always open.

These circumstances I mention the more particularly, as they led to the detection of a similar imposture which was practised by a girl in Germany, about twelve years ago, and of which a minute relation has been furnished by Gruner.* Like

---

* Erzählung der Betrügerei eines Wundermädchens, 12mo. Berlin, 1800. That such impostures were by no means uncommon in former ages, will be abundantly evident from the following list of publications which I extract from Sprengel and Haller.

*Poggii* Florent. de puella Germanica quæ biennium fere vixerat absque cibo potuque. 4to. Florent. 1551.

*Ger. Bucoldiani* brevis enarratio de puella, quæ sine cibo et potu per aliquot annos in pago Roed egit. 8vo. Paris, 1542.

*Catherinæ Binder* inedia. 8vo. Heidelb. 1584.

*Pasc. Rollin* histoire memorable d'une fille d'Anjou, laquelle a etée quatre ans sans user d'aucune nourriture, que d'un peu d'eau commun. 12mo. Paris, 1587.

*Franc. Citesii* Abstinens Confolentana. 8vo. Genev. 1602.

*Pauli Lentuli* historia admiranda Apolloniæ Schreieræ, 4to. Bernæ, 1604.

*J. Chifflet,* asitia in puella Helvetica mirabilis, 8vo. Vesunt. 1610.

*Philip. Sechtleni,* lapis lydius inediarum prodigiosarum, quas plurimi in multos dies, septimanas, menses, et annos protraxerunt, 12mo. Paderborn, 1614.

*Magni Gabr. Block,* Betänckiande öfwr Ester jonodottes longwariga fastande i skone. 8vo. Stockholm, 1719.

*Joh. Doebel* vollständiger Bericht von Ester Johannsen und ihrem langweiligen zehnjahrigen fasten. Hall. 1724.

*C. J. Lossau* Beschriebung eines Casus inediæ. 4to. Hamburg, 1729.

*Charles Fontenelle* sur une fille de Grenoble, que ne boit ni ne mange depuis quatre ans. 4to. Poitiers, 1737.

One of the above mentioned writers (Block) makes an observation which

Like Ann Moore, this girl submitted to a watching of two or three weeks; and like her, too, she passed the ordeal with her integrity unimpeached. If I remember right, she was even subjected to it a second time, without any discovery taking place. But, some time afterwards, a by-stander observing that her linen was generally in a damp state, and had an urinous smell, (to conceal which she used to have heated stones introduced into her bed, under pretence of cold at the pit of her stomach, and would never allow the window to be shut, affecting to faint the moment it was closed,) was led to a train of investigation, which ended in the complete exposure of the various artifices to which she had resorted for the concealment of her evacuations.

11. *Her saying, "that she believes a time may come when God will permit her to eat."*—She is thus prepared for any discovery that may take place, and can never be surprised at her meals or potations without a ready excuse.

12. *The acknowledged fact that she is now in the same, or nearly the same, condition of body as when she commenced her alleged fast.*—"It is apparent," says Mr. Granger,* "that abstinence *qua* abstinence has no effect upon her system. The existence of the patient, after having fasted two years, with a countenance not far removed from the appearance of health, will not be looked upon as an effect of abstinence. For many months together, no wasting is observed." Nay, what is more extraordinary, if the descriptions which have been given of her person by Mr. Granger and others be correct, it will follow, that latterly she must have increased considerably in bulk, for we did not find the abdomen "so remarkably sunk in" as Mr. G. saw it; we did not succeed, like Mr. Taylor, in tracing, with the finger, the grand trunk of the aorta "from the place most immediately under the ensiform process of the sternum, where the loose integument is drawn upon it, nearly to its bifurcation," in drawing it "from its situation over the spine," and "holding the skin across it," so as to shew "both its shape and pulsation;"† nor did Ann Moore appear to us, as she did to J. L. "the most emaciated creature that ever existed."‡ Now it has been shewn that a considerable evaporation is constantly taking place from her lungs and skin; nothing, therefore, which is not unworthy of attention, viz. that all the examples of extraordinary fasting have been confined to the female sex. The remark still holds true with respect to the marvellous cases.

* Edinburgh Medical Journal, vol. v. page 321.

† Medical and Physical Journal, vol. xx.

‡ Loc. cit. page 25.

short short of an actual miracle, can solve the problem of her increased size of body under such circumstances.

13. *The variations and contradictions in her statements.*—It would appear, from Mr. Taylor's narrative, that she continued the use of solid food in small quantities till the end of June 1807; but to us she positively averred that she had taken nothing since the week before Easter of that year. When Mr. Thompson saw her, she acknowledged that she passed a small quantity of urine once a week, and he understood this to be the case at the time when he wrote (August 1809); but to us she declared that she had voided none since the end of August 1808. In reply to a question by Mr. Corn,* she asserted that she *never perspired;* but to us she admitted that she perspired freely when she had fits. To the gentleman whose thermometer she demolished, she complained of pain, and cried out upon the slightest touch of the abdomen; while she allowed us to use considerable pressure without expressing the least uneasiness. On the contrary, she repeatedly assured us that we gave her no pain by the force which we employed.

14. *The inconsistency of her actions with her statements.*—If the attempt to eat and drink really caused her such "misery of deglutition," as Mr. Taylor expresses it, why did she do either, especially as she had lost all desire of food so early as November 1806. Her deceit respecting the contraction of the middle finger of her left hand, and inability to use it, has been already noticed. To Mr. Thompson she pretended to be in a state of such weakness, as made it great labor and even pain for her to attempt to move; but, upon his threatening her with a repetition of the watching, "she so completely forgot her situation," says Mr. T. "that she raised herself upright in bed, a position in which, we had *previously* learned, she had not been for more than a year, griped her fists, threw her arms and head about with as much strength and ease as the most healthy woman of an equal age could possibly do, and talked at the same time most loudly and incessantly, from the effect of violent passion." These inconsistencies alone are sufficient to throw discredit on every thing she says.

Other facts and arguments might be adduced, but I trust that I have collected a sufficient body of evidence to prove that there are no solid grounds for supposing that the order of nature is subverted in the person of Ann Moore; but, on the contrary, that there is every reason to believe that her

* Monthly Magazine, Oct. 1811.

abstinence abstinence is feigned, and that her sufferings are, in a great measure, simulated. Such an inference Mr. Granger is pleased to denominate *an hypothesis;* but the hypothesis appears to me to be on the part of those who imagine that a human being can exist, in a state of comparative health and strength, during a term of months and years, without any of the ordinary means of nutrition; and the only wonder remaining in my mind is, that medical men should be found to lend their sanction to such extraordinary attestations as we have seen concerning the case in question.

*Golden-square,* A. HENDERSON.

*Nov. 25th,* 1812.

